# Divergence across mitochondrial genomes of sympatric members of the *Schistosoma indicum* group and clues into the evolution of *Schistosoma spindale*

**DOI:** 10.1038/s41598-020-57736-x

**Published:** 2020-02-12

**Authors:** Ben P. Jones, Billie F. Norman, Hannah E. Borrett, Stephen W. Attwood, Mohammed M. H. Mondal, Anthony J. Walker, Joanne P. Webster, P. R. V. Jayanthe Rajapakse, Scott P. Lawton

**Affiliations:** 1 Molecular Parasitology Laboratory, School of Life Sciences, Pharmacy & Chemistry, Kingston University London, Kingston Upon Thames, Surrey, KT1 2EE UK; 2 Department of Life Sciences, Natural History Museum, Cromwell Road, London, SW7 5BD UK; 3 Department of Parasitology, Faculty of Veterinary Science, Bangladesh Agricultural University, Mymensingh, 2202 Bangladesh; 4 Centre for Emerging, Endemic and Exotic Diseases, Department of Pathobiology and Population Sciences, The Royal Veterinary College, University of London, Hatfield, Hertfordshire AL9 7TA United Kingdom; 5 Faculty of Veterinary Medicine and Animal Science, Department of Veterinary Pathobiology, University of Peradeniya, Peradeniya, 20400 Sri Lanka

**Keywords:** Molecular ecology, Phylogenetics, Parasite evolution

## Abstract

*Schistosoma spindale* and *Schistosoma indicum* are ruminant-infecting trematodes of the *Schistosoma indicum* group that are widespread across Southeast Asia. Though neglected, these parasites can cause major pathology and mortality to livestock leading to significant welfare and socio-economic issues, predominantly amongst poor subsistence farmers and their families. Here we used mitogenomic analysis to determine the relationships between these two sympatric species of schistosome and to characterise *S. spindale* diversity in order to identify possible cryptic speciation. The mitochondrial genomes of *S. spindale* and *S. indicum* were assembled and genetic analyses revealed high levels of diversity within the *S. indicum* group. Evidence of functional changes in mitochondrial genes indicated adaptation to environmental change associated with speciation events in *S. spindale* around 2.5 million years ago. We discuss our results in terms of their theoretical and applied implications.

## Introduction

*Schistosoma* blood flukes infect over 240 million people and at the very least 165 million cattle worldwide^1,2^. In the mammalian host the parasite causes chronic disease that leads to wasting, anaemia, fibrosis and inflammatory enlargement of internal organs and even cancer^2^. Yet, despite their socio-economic importance, species within the *Schistosoma indicum* group from Southeast Asia are amongst the most neglected of all the schistosomes^3^. The *S. indicum* group contains three species: *Schistosoma spindale*, *Schistosoma indicum* and *Schistosoma nasale*, all of which utilise the snail *Indoplanorbis exustus* as an intermediate host and are widespread across Southeast Asia from India through to the Malay Archipelago^4^. These schistosome species primarily infect bovids, including domestic cattle and water buffalo, and in endemic areas prevalence can be as high as 100%^4^.

*Schistosoma spindale* and *S. indicum* are considered to be sympatric, often occurring in mixed infections and both causing hepato-intestinal schistosomiasis resulting in reduced milk yield, wasting, as well as liver fibrosis due to the occurrence of granulomas around trapped parasites eggs^4^. Across the Indian subcontinent these parasites can cause severe mortality, with outbreaks leading to high death rates in herds^4^. Also, species within the *S. indicum* group are a major cause of human cercarial dermatitis, as a result of the cercaria penetrating the skin, which has become a significant public health problem for people living in endemic regions^5^. Furthermore, experimental evidence has shown that schistosomula can migrate to the lungs or central nervous system in ‘incompatible’ mammalian hosts causing severe pathologies beyond cercarial dermatitis^6^.

Molecular studies have been of great importance in the study of schistosomes, providing vital new information on species identification, population movement, introgression and monitoring of the impact of mass drug administration. Such studies have aided in the focusing of limited resources available for control^7–9^. However, few molecular studies have been undertaken on members of the *S. indicum* group, with the majority focused primarily on species identification and phylogenetics using mitochondrial markers, particularly the species barcoding gene the cytochrome c oxidase subunit 1 (*cox1*), the *16* *s* and *12* *s* ribosomal subunit RNA^3,10–12^. To date, Attwood *et al*.^10^ and Devkota *et al*.^11^ have provided the most detailed account of the evolution of *S. indicum* group using phylogenetic comparisons to illustrate the complexity of the radiation of the group across Southeast Asia and noting the high levels of diversity of *S. spindale*. Devkota *et al*.^11^ also utilised *cox1*, *16* *s* and *12* *s* to identify schistosome species parasitizing *I. excutus* from Nepal, demonstrating a division between *S. spindale* populations and a potentially undescribed species which was referred to as *Schistosoma cf. indicum*. The study highlighted the need for continued molecular epidemiological surveys of the *S. indicum* group but also showed that the group is more diverse than originally thought which could account for heterogeneity in infection and pathology^11^.

Over the past decade, full mitochondrial genomes have been used for comparative analysis within and between species, predominantly in the *Schistosoma mansoni*, *Schistosoma haematobium* and *Schistosoma japonicum* groups^13–15^. However, only a single complete mitochondrial genome has been published for *S. spindale*, using specimens from Sri Lanka which resolved the relationship between the *S. spindale* and *S. haematobium* groups^15^. In this current study we aimed to assess the differentiation between the sympatric parasites *S. indicum* and *S. spindale* by sequencing and comparing complete mitochondrial genomes of isolates from Bangladesh with that of the published mitochondrial genome of a *S. spindale* isolate from Sri Lanka. Furthermore, we assessed the divergence between isolates of *S. spindale* to clarify the level of differentiation between different populations and identify the suspected occurrence of cryptic species^11^. Complete mitochondrial genomes of *S. spindale* from Bangladesh and Sri Lanka were compared to provide genomic insights into the divergence between the north and the south of the Indian subcontinent. We predicted that, overall, the two new mitochondrial genomes of *S. spindale* and *S. indicum* would show a great deal of similarity to each other in terms of genome size and structure, owing to the close relationship between these species. We hypothesised that there would be high levels of diversity between the new *S. spindale* data from Bangladesh and the reference mitochondrial genome from Sri Lanka, with the new sequences being more similar to samples from Nepal in comparative genes, which could be evidence of cryptic speciation in *S. spindale*.

## Results

### Divergence across the mitochondrial genomes of *S. spindale* and *S. indicum*

**Figure 1:**
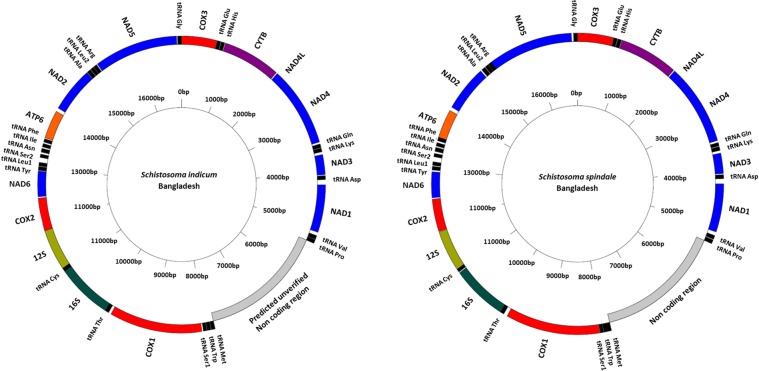
Circularised mitochondrial genomes of *Schistosoma indicum* and *Schistosoma spindale* from Bangladesh. Graphical representations generated by Genome VX.

Using the published Sri Lankan *S. spindale* mitochondrial genome (accession: DQ157223) as reference, complete mitochondrial genomes of *S. spindale* and *S. indicum* (accession: MN637820 and MN637821 respectively) were assembled and annotated, all showing the derived gene order shared with the African *S. mansoni* and *S. haematobium* groups (Fig. 1)^13^. No major differences in the position or size of the genes were seen. Likewise, although no differences were identified between the Sri Lankan and Bangladeshi *S. spindale*, there were, however, eight tRNAs with distinct structures observed in *S. indicum*, which included those carrying the anticodon for cysteine (C), histidine (H), isoleucine (I), asparagine (N), glutamine (Q), arginine (R), tryptophan (W) and tyrosine (Y) (Supplementary Fig. S1).

**Figure 2:**
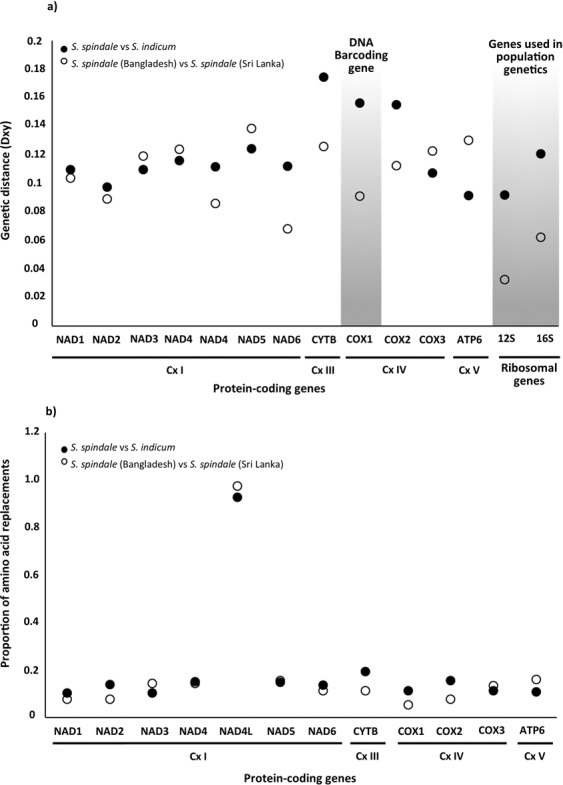
Nucleotide divergence and amino acid replacements in mitochondrial genes between *S. indicum* and *S. spindale*, and isolates of *S. spindale*. Where (**a**) is nucleotide divergence based on pairwise genetic distance calculated as the average number of nucleotide substitutions per site (Dxy) with blocked areas highlighting the species identification cox1 gene and the ribosomal genes used in population genetic studies. (**b**) is the proportion of amino acid differences calculated as the total number of replacements within each protein sequence divided by the protein length. The oxidative phosphorylation system complex (Cx) is highlighted.

Divergence between mitochondrial genomes was assessed by calculating pairwise averaged nucleotide divergence (Dxy) for each of the individual protein coding and ribosomal coding genes. Comparisons were performed between both *S. spindale* isolates with *S. indicum*, alongside direct comparisons between the Bangladeshi and Sri Lankan *S. spindale*. The cytochrome b (*cytb*) gene had the highest levels of Dxy at 0.17461, with the ATP synthase F0 subunit 6 (*atp6*) gene showing the lowest level of Dxy at 0.09162 when comparing *S. spindale* to *S. indicum* (Fig. 2a). Notably, the cytochrome oxidase subunit 1 (*cox1*) gene appeared to have the second highest Dxy at 0.15652, all other genes had a Dxy within a narrow divergence range of 0.10752–0.12445. Unlike the *12* *s* ribosomal gene, which displayed low Dxy at 0.09211, the *16* *s* gene had a Dxy of 0.12081 comparable to that of the other protein coding genes such as the NADH dehydrogenases (*nad1-6*) (Fig. 2a). Although, the inter *S. spindale* comparisons showed lower levels of Dxy, the distribution of divergence across the mitochondrial genome was different with *nad5* showing the highest Dxy at 0.13863. Unlike the interspecies comparisons, *cox1* also showed low levels of Dxy at 0.09127. However, the ribosomal genes had the lowest levels of Dxy with *16* *s* at 0.0625 and *12* *s* at 0.03289 in the inter *S. spindale* comparison (Fig. 2a). In order to account for the impact of such divergence on genes and their resultant proteins the total number of amino acid replacements per gene was estimated for comparisons of both *S. spindale* and *S. indicum* as well as between *S. spindale* from Sri Lanka and Bangladesh. Although the inter-species comparisons showed a generally higher rate of proportional amino acid replacements of 0.106–0.195 than the inter *S. spindale* comparison at 0.056–0.161, both comparisons showed *nad4L* to have the highest proportion of amino acid replacement (Fig. 2b).

**Figure 3:**
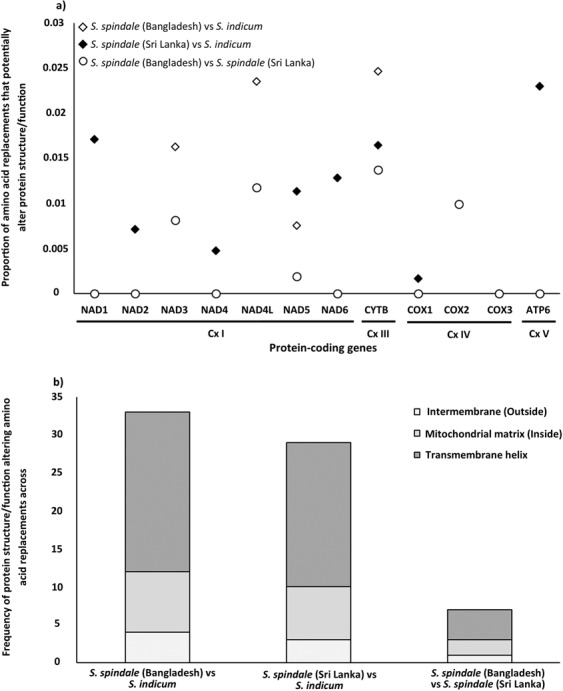
Proportion of amino acid changes with functional effects on mitochondrial proteins and the frequency and locality of replacements across the protein in comparisons between *S. indicum* and *S. spindale*, and isolates of *S. pindale*. Where (**a**) is the proportion of amino acid replacements identified to have a potential functional change to the resultant protein as predicted by Provean^26^. (**b**) is the frequency of potential functional changing replacements found within the different domains of the mitochondrial proteins as identified by TMHMM^16^ searches.

Owing to the small number mitochondrial genomes being compared in this study it was not possible to use standard population genetic methods to detect the occurrences of positive selection on each of the genes within and between species. Nevertheless, to account for the levels of amino acid replacements seen across the mitochondrial genomes indirect assessment of selection were performed by assessing the potential impact of amino acid changes and identifying the regions within the proteins that these mutations occur. Provean^15^, protein variation effect analyses, identified a total of 33 functionally altering amino acid replacements across nine proteins when the Bangladeshi *S. spindale* was compared to *S. indicum*, and 29 in Sri Lankan *S. spindale* and *S. indicum* comparison (Supplementary Table S1). However, only nine functionally altering amino acid replacements were found across five proteins when the Bangladeshi and Sri Lankan *S. spindale* were compared (Supplementary Table S1). Overall, when the proportion of functionally altering amino acid changes were calculated per gene, *cytb* showed the highest proportion of functional mutations followed by *nad4L* in comparisons between *S. spindale* and *S. indicum* and between the *S. spindale* isolates (Fig. 3a). Also, the NADH dehydrogenases appeared to have the highest proportion of function altering mutations relative to other genes excluding those discussed above. Interestingly, the *cox1* gene showed the lowest level frequency of function altering amino acid replacements between *S. indicum* and *S. spindale*, and none were detected in the intra-species comparisons (Fig. 3a). The TMHMM^16^ analyses indicated that the majority of the functional altering amino acid replacements occurred within the transmembrane domains of the proteins and not within the functional domains exposed on either the outer or inner surface of the mitochondria (Fig. 3b). However, in the comparisons of *S. spindale* and *S. indicum* there were two amino acid replacements in the region of the protein exposed to the mitochondrial matrix inside the mitochondria in cytochrome oxidase subunit 2 protein (*cox2*), three found in *cytb*, two in *nad1*, one in *nad5* and a further two in *nad1*. Such replacements were not as extensive in the inter *S. spindale* comparisons with only four alterations being found in the domains exposed to the mitochondrial matrix, two of them in *cox2* and the others in *cytb* (Fig. 3a,b; Supplementary Table S1). Fewer alterations were found within domains of the protein exposed to the surface of mitochondria. When *S. spindale* and *S. indicum* were compared, only four such alterations were identified, a single alteration in *cox1*, *cytb*, *nad4* and *nad5*. Notably, in the inter *S. spindale* comparisons a single alteration was found in *cytb* (Supplementary Table S1).

### Phylogenetic reconstruction of the *S. indicum* group

**Figure 4:**
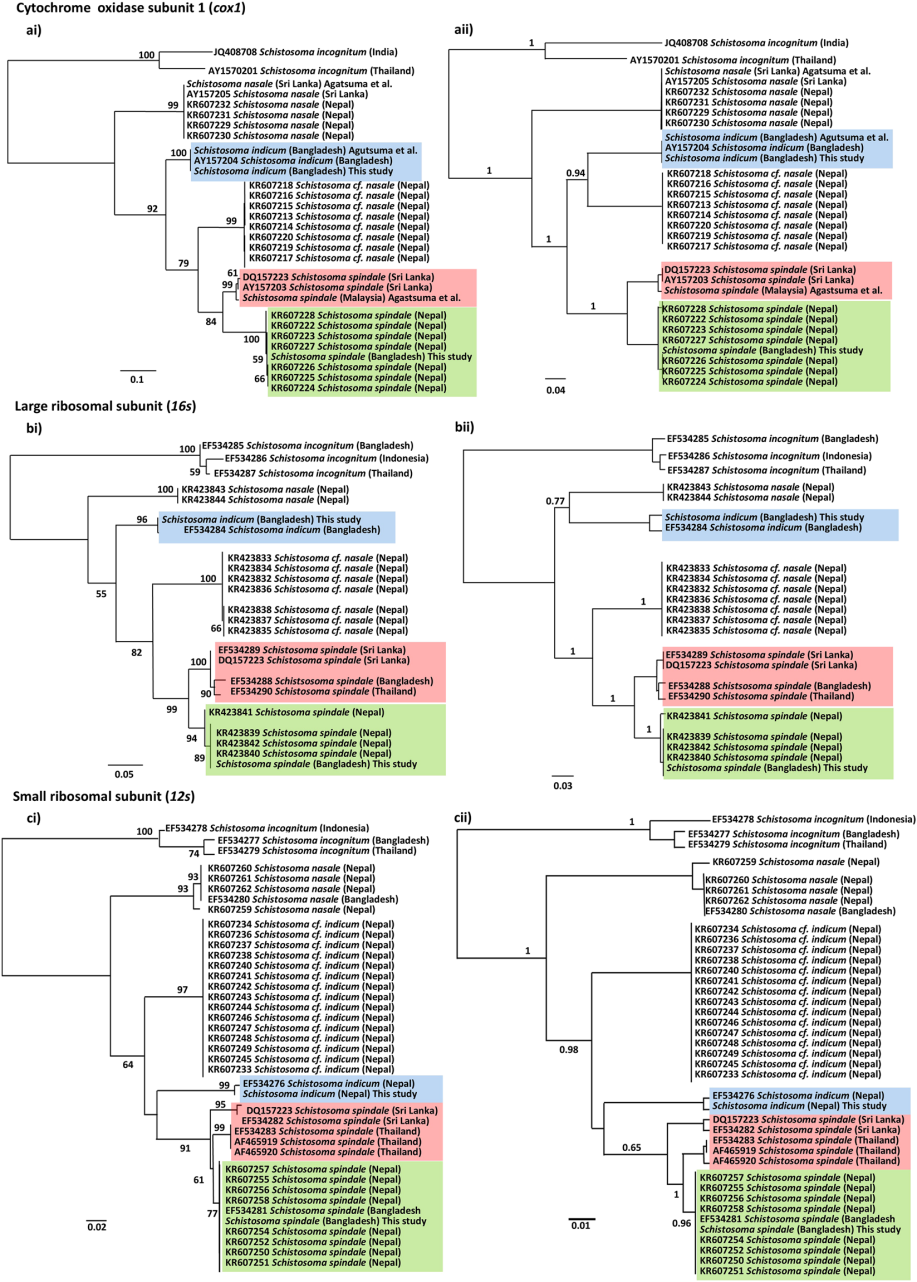
Maximum likelihood and Bayesian inference phylogenies of mitochondrial genes from the *Schistosoma indicum* species group. *Schistosoma incognitum* was employed as the outgroup. Were (**a**) are phylogenies constructed using the *cox1* gene using the HKY + G + I model for both ML (**ai**) and bi (**aii**) analyses; (**b**) are phylogenies constructed using the *16 s* ribosomal sequence using the GTR + G model for both ML (**bi**) and bi (**ii**) analyses; (**c**) are phylogenies constructed using the *12 s* ribosomal sequence HKY + G for both ML (**ci**) and bi (**cii**) analyses. Nodal support values are provided by 1000 bootstrap replicates in the ML analyses and posterior nodal values for the bi analyses. The position of *Schistosoma indicum* is highlighted in blue, with the *Schistosoma spindale* cluster from Sri Lanka/Thailand/Malaysia being highlighted in pink, and the *S. spindale* cluster from Nepal/Bangladesh highlighted in green.

Owing to the paucity of comparable mitochondrial genomic data for the *S. indicum* group, *cox1*, *16* *s* and *12* *s* gene markers were employed to provide a phylogenetic perspective of the group, consistent with that employed to date across the majority of published studies and hence the largest comparable data sets available (Supplementary Tables S2–S4). Initial calculations of phylogenetically informative sites showed the *cox1* to have 299 out of 985, the *16* *s* 237 out of 632, and the *12* *s* 76 out of 333 and subsequent maximum likelihood (ML) phylogenetic reconstruction (Fig. 4ai–ci) showed similar overall topologies with well supported clades with nodal bootstrap values > 50 for each of the gene markers. Each phylogeny showed *S. nasale* to be the most basal within the group and *S. spindale* the most derived taxa. In all phylogenies there was a clear separation between *S. cf. indicum* and *S. indicum*. Topologies of the *cox1* and *16* *s* phylogenies appeared identical with *S. cf. indicum* resolving as an intermediate between *S. spindale* and *S. indicum* (Fig. 4ai–bi). However, in the *12* *s* analyses *S. cf. indicum* appeared as a paraphyletic sister taxon to an *S. indicum* and *S. spindale* clade. Clear distinction was identified between the *S. spindale* populations which split into two well supported clades with each tree producing a distinct Nepal/Bangladesh and a Sri Lanka/Thailand/Malaysia cluster, with the exception of the *16* *s* phylogeny which showed a single sequence from Bangladesh within the Sri Lanka/Thailand/Malaysia lineage. Although phylogenetic trees constructed using Bayesian inferences (BI) produced well supported phylogenies with high posterior nodal support values and broadly illustrated the same over relationships as the ML analyses, only the *12* *s* phylogenetic topologies were identical between analyses (Fig. 4ci,cii). There were notable differences in the relationships between *S. indicum* and *S. cf. indicum*, and unlike the *cox1* and *16* *s* ML phylogenies, the BI analyses resolved *S. indicum* in different phylogenetic positions, showing *S. indicum* to be a sister taxa to *S. cf. indicum* in the *cox1* phylogeny, with the *16* *s* showing *S. indicum* to share a distinct clade with *S. nasale* (Fig. 4ai and ii,bi and ii). Similarly, the *16* *s* BI analyses also showed the *S. cf. indicum* to be a sister taxa to *S. spindale*. The BI phylogenies showed the same relationships between *S. spindale* isolates as the ML analyses across all three markers with the prominent two major clusters occurring as described above (Fig. 4a–c).

**Figure 5:**
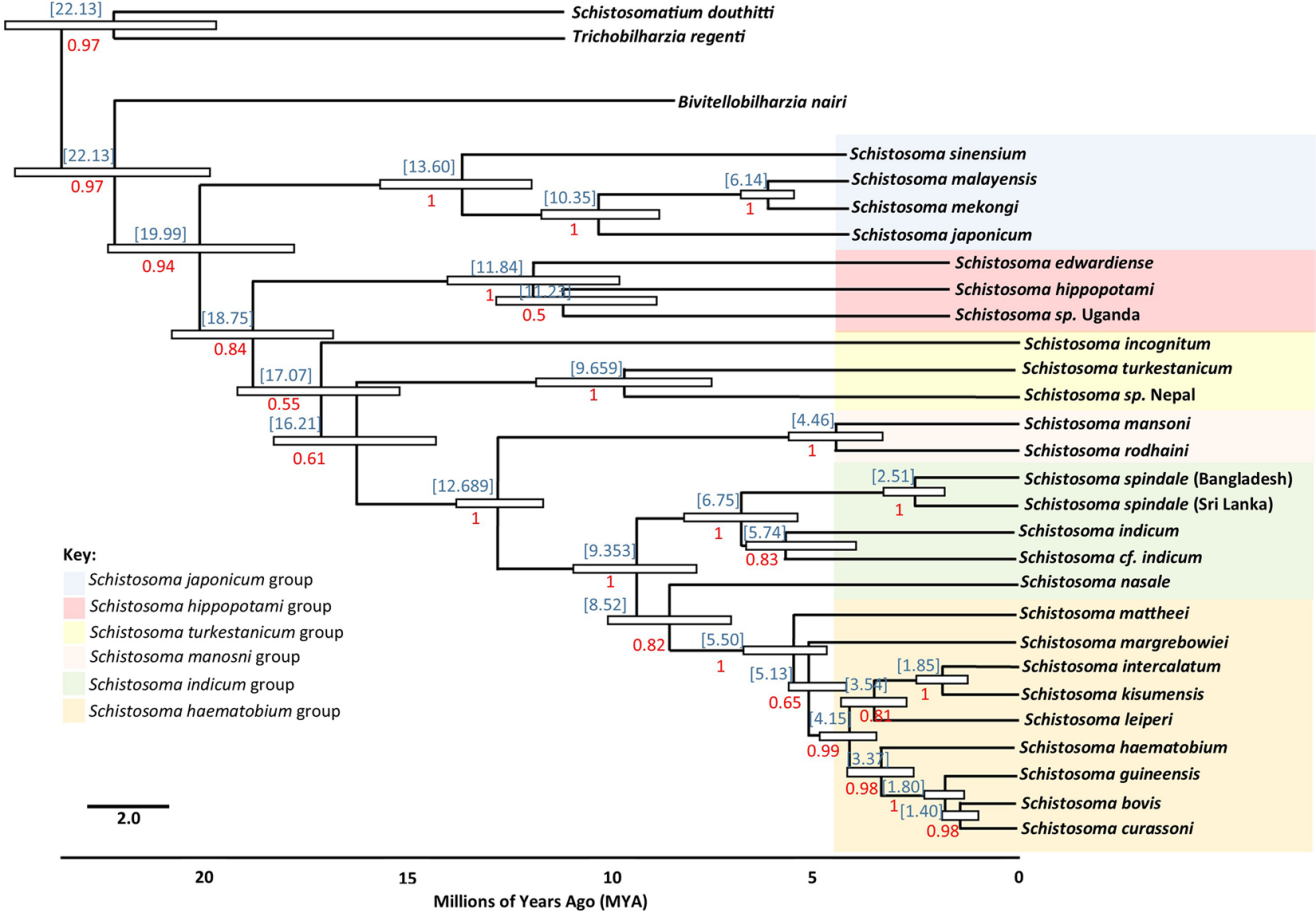
*Schistosoma* Bayesian timetree based on molecular clock analyses. Molecular clock created in BEAST showing the mean time of most recent common ancestor (MRCA) for all available schistosome species (blue) and the posterior probabilities (red). The white bars at each node show the highest 95% posterior density (HPD) interval for the main nodes an indicator of the estimated range of time of divergence. Molecular clock predicts divergence of the *S. indicum* group at 9.35 MYA. This molecular clock predicts the *S. indicum* group to be paraphyletic with *S. nasale* as the most derived of the group and separate from the other species. The MRCA between the two populations of *S. spindale* is predicted to be approximately 2.51 MYA.

Due to the low levels of divergence identified within Nepal/Bangladesh and Sri Lanka/Thailand/Malaysia clades, single representation of *cox1* sequences were used from the complete mitochondrial genomes of *S. spindale* from Sri Lanka and Bangladesh, along with those from *S. indicum*, in order to estimate divergence times within the *S. indicum* group using BEAST^17,18^. Owing to the lack of sequence data for the *S. indicum* group, a range of schistosome species were used to aid the estimation of divergence times across *Schistosoma* available *cox1* data (Supplementary Table S5), as well as sequences for *Bivetellobilharzia nairi*, *Schistosomatium douthitti* and *Trichobilharzia regenti*, the latter of which were used as the outgroup. The *cox1* marker has been historically used to calculate divergence time between schistosome species and populations, and the use of other *Schistosoma* species allowed the calibration of the clock analyses using predicted divergence times between *S. mansoni* and *S. japonicum*, *S. incognitum* and *S. mansoni*, and *S. mansoni* and *S. haematobium* as highlighted by Lawton *et al*.^19^. This approach allowed time to the most recent common ancestor (TMRCA) for each node to be calculated across the schistosome phylogeny. Effective sample size (ESS) was in excess of the 200 thresholds, providing reliable posterior probabilities and a greater level of statistical certainty of estimated divergence times^17,18^. The Bayesian timetree produced by BEAST provided the accepted standard phylogenetic topology of the schistosomes as highlighted Lawton *et al*.^19^. However, in the time tree *S. nasale* appears as a sister taxon to the *S. haematobium* group rather than clustering with the rest of the *S. indicum* group as also seen in the cox1 phylogeny produced by Devkota *et al*.^11^. Thus, the clock is considered only to be a conservative estimate of divergence time for the group and predicts the emergence of the ancestor of the *S. indicum* group approximately 9.35 million years ago (MYA). The timetree resolved *S. spindale* and *S. indicum* as sister taxa and estimated their divergence at approximately 6.75 MYA with *S. indicum* from Bangladesh and *S. cf. indium* from Nepal predicted to have diverged from each other approximately 5.74 MYA. However, the divergence of the two *S. spindale* lineages was estimated to have occurred much more recently around 2.51 MYA (Fig. 5).

### Divergence in *schistosoma spindale*

**Figure 6:**
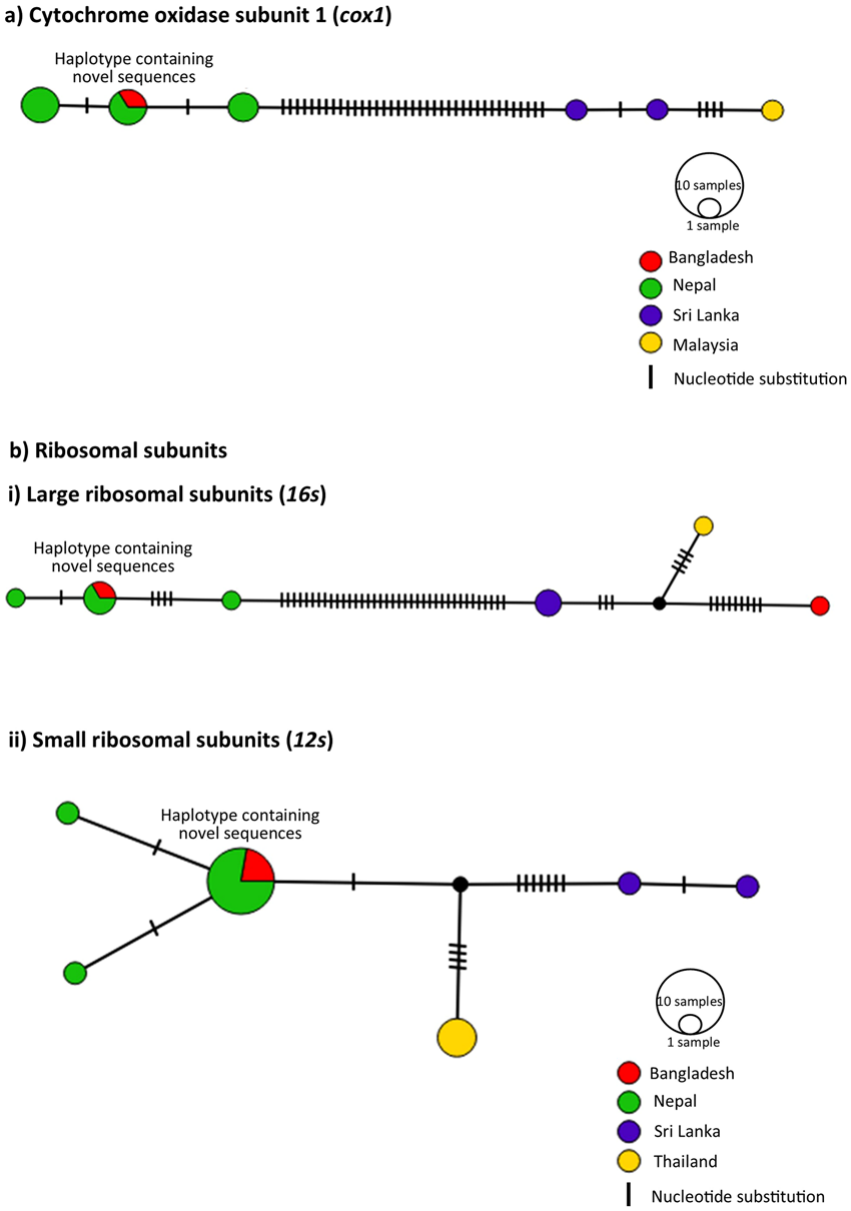
Population structuring of *S. spindale*. Haplotype networks created in PopART using TCS network method, with country of origins represented as colours and size of circle indicating number of sequences in single haplotype. Dashes represent single mutations.

Haplotype network analyses of *S. spindale* mirrored the relationships identified in the phylogenetic analyses, where the *cox1* and *16* *s* showed two distinct clusters, the first comprising of haplotypes from Nepal and Bangladesh, with haplotypes being shared between both localities (Figs. 4a,b and 6a,b). The other cluster contained sequences from Sri Lanka and Thailand or Malaysia, but within the *16* *s* haplotype network a single sample from Bangladesh clustered with the Sri Lankan and Thai samples. Despite the occurrence of fewer mutations between sequences, the network produced for *12* *s* showed a similar clustering of the haplotypes but separated Thailand haplotypes from those of Sri Lanka more readily, which was also reflected in the occurrence of separate Sri Lankan, Thai and Nepal/Bangladesh clusters in the ML phylogenetic reconstruction (Figs. 4c and 6c).

Both the *cox1* and *16* *s* networks revealed substantial divergence between the Nepal/Bangladesh and a Sri Lanka/Thailand/Malaysia clusters. In order to account for the occurrence of any potential speciation events, uncorrected *p*-distances between sequences were calculated as a measure of divergence. When all *S. spindale* sequences were assessed, there were elevated levels of divergence based on average uncorrected *p*-distance with *cox1* showing 4.25% and *16* *s* showing 4.27% divergence (Table 1). Despite the occurrence of three separate linages in *12* *s*, the average divergence was relatively low compared to the other markers (1.24%). However, when divergence was compared between lineages, the Nepal/Bangladesh and Sri Lanka/Thailand/Malaysia clusters the *cox1* possessed the highest level of divergence (8.9%) followed by the *16* *s* with a divergence of 7.3% (Table 1).

**Table 1:** Uncorrected P distance calculation within and between *Schistosoma spindale* populations.

	Comparisons	Genes		
		Cox1	16s	12s
Within clades	All	0.0425	0.0427	0.0123
	Bangladesh/Nepal clade	0.0058	0.0056	0.0027
	Sri Lanka/Malaysia clade	0.0092	na	na
	Sri Lanka/Thailand/Bangladesh clade	na	0.0169	na
	Sri Lanka clade	na	na	0.003
	Thailand clade	na	na	0
Between clades	Bangladesh/Nepal clade vs Sri Lanka/Malaysia clade	0.089	na	na
	Bangladesh/Nepal clade vs Sri Lanka/Thailand/Bangladesh clade	na	0.073	na
	Sri Lanka clade vs Thailand clade	na	na	0.032
	Sri Lanka clade vs Bangladesh/Nepal clade	na	na	0.028
	Thailand vs Bangladesh/Nepal clade	na	na	0.016

The data indicates that the *S. spindale* population from Nepal and Bangladesh may be a separate species to the population from the rest of Southeast Asia as there is a high level of distance seen within the species as a whole yet when each population is analysed separately this level drops dramatically and the distance between the populations is comparatively high.

The same pattern was reflected in the automatic barcode gap discovery (ABGD)^20^ analyses used to determine molecular operational taxonomic units as potential species. When assessing the complete data for the *S. indicum* group, five distinct groups were identified within *cox1* and *16* *s* data sets at the species cut-off value of 0.0359 (>3% divergence based on uncorrected *p*-distance) indicating five separate species within the *S. indicum* group: *S. nasale, S. indicum, S. cf. indicum*, with *S. spindale* split into two distinct species groups (Sri Lanka/Malaysia and Bangladesh/Nepal). Conversely, although the *12* *s* resolved differences between *S. nasale*, *S. indicum*, and *S. cf. indicum* it did not separate *S. spindale* into two distinct taxonomic units. In order to validate the findings described above the Birky^21^ 4X ratio was applied to provide a lineage specific perspective of species delimitation rather than depending on average divergence estimations based on gross pairwise differences between DNA sequences. In contrast to the previous analyses, the 4X ratio calculations failed to differentiate *S. spindale* in to distinct species and resolved all sequences from across Southeast Asia as a single species, indicated by all values of K/θ = <4 (*cox1*- 2.45, *16s*- 2.78 and *12s*- from 0.87–1.63) (Table 2).

**Table 2:** 4X rule calculation for species delimitation.

Gene	Cox1		16s		12s					
Clade	Nepal/Bangladesh	Sri Lanka/Malaysia	Nepal/Bangladesh	Sri Lanka/Malaysia	Nepal/Bangladesh	Thailand	Nepal/Bangladesh	Sri Lanka	Thailand	Sri Lanka
n	8	3	5	4	11	3	11	2	3	2
K	0.105		0.065		0.016		0.024		0.03	
d	0.003	0.009	0.003	0.015	0.001	0	0.001	0.003	0	0.003
π = d*(n/n − 1)	0.0034	0.0135	0.0038	0.0227	0.0011	0.006	0.0011	0.006	0	0.006
θ = π/(1 − 4π/3)	0.0104	0.0428	0.0037	0.0233	0.0033	0.0184	0.0033	0.0184	0	0.0184
K/θ	2.451		2.7809		0.8676		1.3013		1.6267	

Calculations used to delimit species under the 4X rule (Birky, 2013) for *cox1*, 16s and 12s show that under a conservative model the two *S. spindale* population represent a single species as all results for K/θ are below 4, with the exception of the 12s populations from Thailand and Sri Lanka. Where n = numbers of sequences; K = total pairwise differences between clades; d = pairwise differences within clades.

In order to account for the cause of the divergence between the *S. spindale* isolates, alterations in physiochemical properties of the *cox1* sequences were identified as an indicator of the impact of codon specific selection across the *S. spindale* phylogeny using TreeSAAP^22^. By utilising a sliding window approach, TreeSAAP^22^ compared the total number of observed amino acid replacements relative to those evolving under neutrality across the *S. spindale cox1* phylogeny. The analyses detects the occurrence of 20 physiochemical properties across eight categories, where 1–3 are considered to have little impact on the resulting protein, 4–5 having medium effect, and 6–8 having a substantial effect, altering the biochemistry of the protein and considered to be evidence of codon specific positive selection. Only seven physiochemical alterations appeared to have occurred within the *cox1* protein, with four possessing z-scores > ±3.09 with *P* < 0.001. The properties which had the most profound effect were in category level 7 and were alterations in isoelectric point (z = 4.248) and short medium range non-bond energy (z = 3.995) (Table 3). Category level 6 alterations were also shown for partial specific volume (z = 4.543) and helical contact area (z = 3.85). This is illustrative of the phenotypic impacts of the mutations between the *S. spindale* isolates and relates the divergence between isolates to potential subtle local adaptive differences. When the same analyses were performed for *S. indicum* and *S. cf. indicum cox1* no significant differences were identified (data not shown).

**Table 3:** Property showing signs of adaptive changes in *Schistosoma spindale cox1*.

Property	Categories (Z-scores)		
	6	7	8
Equilibrium constant (ionization of COOH)			1.792*
Isoelectric point		4.248***	
Short and medium range non-bonded energy		3.995***	
Solvent accessible reduction ratio		2.168*	
Power to be at the C-terminal		−1.78*	
Partial specific volume	4.543***		
Helical contact area	4.385***		

TreeSAAP results for *cox1* analysis of the two *S. spindale* samples reveal that isoelectric point and short and medium range non-bonded energy show the most prominent signs adaptation. Only changes with P < 0.001 (***) are regarded as highly significant and useful in terms of adaptation. These adaptive changes in isoelectric point have been previously linked which adaptive changes to abiotic pressures.

## Discussion

Comparative analyses of the complete mitochondrial genomes of *S. spindale* and *S. indicum* revealed that, although variation and diversity exist, nucleotide divergence (Dxy) is reasonably low between species (Dxy = 0.09–0.17). Interestingly, with the exception of *cox1*, *nad6*, *atp6*, *16* *s* and *12* *s* the intra-species comparison of the Bangladeshi and Sri Lankan *S. spindale* broadly showed the same regions of the mitochondrial genome to display the same patterns of divergence as that seen between *S. spindale* and *S. indicum*. Such patterns have been suggested to reflect the major processes driving species level divergence as illustrated with the *S. japonicum* group^14^ and in comparisons of *S. japonicum* populations between mainland Asia and surrounding Islands^14,23^. However, owing to the sympatric nature of host-sharing in *S. spindale* and *S. indicum*, divergence in such regions of the mitochondrial genomes could be indicative of the establishment of speciation barriers, giving rise to alterations in the function of genes and ribosomal structures especially tRNAs^24,25^. Although it was not possible to assess the occurrence of positive selection across the mitochondrial genomes using standard population genetic approaches in this study due to low sample size, amino acid replacement analyses did illustrate high rates of substitution. Likewise, the Provean^26^ and TMHMM^26^ analyses highlighted a number of potentially function altering amino acid changes in regions of the peptides directly involved in the electron transport machinery of the mitochondria. Although most of the genes required for mitochondrial function are encoded by the nucleus, genes for five enzymatic complexes have been retained in the mitochondria in *Schistosoma*^13^. These complexes include those composed of the genes which regulate oxidative phosphorylation (OXPHOS) processes including complex 1 (Cx I) containing *nad1-6*, complex 3 (Cx III) containing *cytb*, complex 4 (Cx IV) containing *cox1-3*, and complex 5 (Cx V) comprising of the single *atp6* gene responsible for ATP synthesis^27^. In our study, Cx I and Cx III were shown to contain the majority of amino acid replacements potentially affecting protein function, illustrating the signature of positive selection. Although the majority of mutations were found in transmembrane regions of proteins, which may result in subtle alterations in protein shape, the inter-species comparison of *cytb*, *nad1* and *nad4L* showed the highest proportion of function altering changes, with *cytb* and *nad1* possessing mutations in the protein region directly within the mitochondrial matrix, potentially affecting the efficacy of the electron transport chain. Several studies have shown similar patterns of mutation in Cx I and Cx III invertebrates that have been linked to not only local environmental adaptation within species but also the establishment of cyto-nuclear incompatibilities between closely related sympatric species and ultimately causing hybrid break down^24,25^.

Ribosomal genes have also been implicated in translational deficiency in hybrids, as nuclear ribosomal subunits required for their translation are highly divergent between species. This results in the inability of accurate transcription and translation of the mitochondrial ribosomal subunits and tRNAs, with the subsequent failure of mitochondrial function in hybrid offspring^25^. Such a process could account for the alternative tRNA structures observed between *S. spindale* and *S. indicum*; tRNA function is structure-dependent and single nucleotide mutations in the anticodon loops of mitochondrial tRNAs can reduce OXPHOS capacity owing to reduced translational efficacy in hybrids due to species specific mutations^28,29^. Hybridisation events among schistosomes are becoming well documented, however, there are no detailed studies of the phenomenon within the *S. indicum* species group^30^. Despite their sympatric nature, inter-species mating between *S. spindale* and *S. indicum* and the production of viable hybrid eggs have been recorded only under laboratory conditions^31^. Therefore, alterations within the structure of the tRNAs alongside the divergence within the OXPHOS Cx I and Cx III, may be indicative of the development of initial cyto-nuclear speciation barriers between *S. indicum* and *S. spindale*. However, it is important to note that in order to truly disentangle the mechanism of speciation processes in the *S. indicum* group, increased sampling and genome sequencing of *S. indicum* and *S. spindale* from a range of geographical locations across Asia is essential.

Speciation across the *Schistosoma* genus is highly complex with little known about the processes and mechanisms affecting inter-species divergence or intra-species. The phylogenetic analyses here support that of Devkota *et al*.^11^ showing clear distinctions between *S. indicum* and *S. cf. indicum*, as well as between populations of *S. spindale*. With the exception of the BI *16* *s* phylogeny all other analyses showed *S. nasale* to be basal within the *S. indicum* group forming a paraphyletic sister clade to the closely related *S. indicum*, *S. cf. indicum* and their sister taxa *S. spindale*. Close evolutionary relationships between the *S. indicum* and *S. haematobium* groups appears to be associated with both groups having evolved to infect ungulates. The molecular clock predicted the divergence of the *S. indicum* group in the late Miocene, approximately 9.35 MYA, substantially earlier than previous predictions of approximately two million years ago during the major mammal migration from Africa to Asia^10^. This coincides with a major change in plant flora across Africa as a result of reductions in temperature, which lead to the domination and radiation of bovids around 7–15 MYA^32^. Interestingly, Gauffre – Autelin *et al*.^33^ indicated that *Indoplanorbis* is also most likely to have an African origin and diverged from *Bulinus*, the intermediate host for the *S. haematobium* group, in the early Miocene, 17–38 MYA. *Indoplanorbis* would have then undergone a dispersal along the middle eastern land connection before radiating across Asia from the mid to late Miocene 5–15 MYA. This *Indoplanobis* dispersal event coincides with the mass movement of animals back and forth between Africa and Eurasia^32^ providing a potential mechanism for the proto-*S. indicum* group to leave Africa and disperse into Asia. The molecular clock analyses predicted the divergence of *S. spindale* and *S. indicum* at approximately 6.74 MYA with *S. indicum* and *S. cf. indicum* diverging approximately 5.74 MYA The divergence event between *S. spindale* and *S. indicum* coincides with the late Miocene period of tectonic and climatic instability; during this period there would have been a rapid uplift of the Qinghai–Tibetan Plateau which would have initiated and subsequently intensified the monsoons. This event would have isolated several bovid species carrying ancestral parasite populations giving rise to *S. spindale* and *S. indicum*. Again, the timing of this radiation event coincides with a major diversification event of *Indoplanorbis* across Asia^33^. It could be suggested that proto-*S. indicum* would have become isolated on the Indian subcontinent and rapidly radiated across the continent giving rise to *S. indicum* in India, Bangladesh and Pakistan and *S. cf. indicum* in Nepal. This may account for the specificity of *S. indicum* on the Indian subcontinent and its absence elsewhere across South East Asia, but it is clear that there is a direct association between the movement of definitive bovids hosts, the radiation of *Indoplanorbis* as an intermediate host and the diversification of the *S. indicum* group.

Comparisons between *S. spindale* from Bangladesh and Sri Lanka here showed distinct nucleotide divergence across the mitochondrial genome with some genes showing similar levels of divergence as seen between *S. spindale* and *S. indicum*. Similarly, both phylogenetic and haplotype network reconstruction highlighted clear separation between *S. spindale* populations producing Nepal/Bangladesh and a Sri Lanka/Thailand/Malaysia clusters. When several methods of species delimitation were applied to the data mixed results were obtained. The ABGD analyses indicated the presence of two species and *p*-distance calculations favoured patterns that indicate speciation in *S. spindale* with intraspecific divergence below 1% and interspecific divergence around 10X that of intraspecific^34^. Conversely the K/θ ratio method failed to predict separate species^21^. Such divergence between populations has been shown to occur in other animal groups and is considered to represent evidence of a speciation event in action^35–38^. These patterns are the result of vicariance where populations have become isolated as a result of a geographical barrier, leading to rapid speciation, which can be followed by secondary contact of populations before full speciation barriers have arisen^35–38^. The molecular clock estimated divergence between the two clusters of *S. spindale* in the late Pliocene-early Pleistocene, around 2.5 MYA, a time when sea levels were lower and most of the Southeast Asian islands were part of the continental landmass^39^. This may account for the close relationship between parasites from the Sri Lanka, Malaysia and Thailand, as the ancestral population would have moved freely across the region with their hosts^40^. The timing of this divergence also coincides with the last major uplift of the Himalayas, of between 2200–3400 metres since the Pliocene, providing the vicariance event required to separate the *S. spindale* populations^41^. However, it is important to consider that the relationships between *S. spindale* populations within the Sri Lanka/Thailand/Malaysia cluster may in fact result from recent invasion events due to the movement of cattle between these regions as a result of human migration and trade in livestock^10^. This could account for the *16* *s* Bangladeshi haplotype which fell within the Sri Lanka/Thailand/Malaysia cluster and the general lack of diversity.

Although substantial amino acid replacements were identified across the peptides produced by mitochondrial genes when the *S. spindale* isolates were compared, only eight replacements across four genes appeared to have any potential functional effect based on the Provean and TMHMM analyses. As with the inter-species comparison, genes within Cx I and Cx III appeared to have the highest number of functional replacements with *cytb* having the highest proportion of such mutations overall. Replacements were found in both regions of the *cytb* proteins that directly interact with the internal matrix and those that are exposed to the intermembrane on the surface of the mitochondria. Again, owing to the high level of conservation in the *cytb* protein due to its functional role in creation of proton gradient and electron transfer to Cx IV mutation in this protein would have profound effect of the efficiency of the respiration processes^42^. Interestingly, the *cox 2* gene of Cx IV also showed functional amino acid changes in its peptide in the regions also exposed to the internal mitochondrial matrix. Such alterations have been shown to reduce coupling efficiency of the OXPHOS across the electron transport chain in other animals and have been linked to adaptations to colder climates^42,43^.

Although not detected by the Provean^26^ analyses conducted on the genes from the complete mitochondrial genomes, when *cox1* was compared between the Nepal/Bangladesh and Sri Lanka/Thailand/Malaysia clusters of *S. spindale* substantial nucleotide differences were identified, several of which were non-synonymous. TreeSAAP^22^ revealed a number of profound changes in physiochemical properties including isoelectric point, which has been linked with adaptation to low temperature or high-altitude environments^44–49^. Alterations in isoelectric point can cause changes in the folding of the cytochrome oxidase subunit proteins, resulting in proton leakage making the OXPHOS pathway less efficient^50^. Both analyses from the complete mitochondrial genomes of *S. spindale* and the inter population variation analyses of the Nepal/Bangladesh and Sri Lanka/Thailand/Malaysia clusters are indicative of adaptation to colder climates or hypoxic environments, as a less efficient OXPHOS pathway results in increased metabolic heat^47^, and increases expression of hypoxia specific genes^49,51^. Such adaptations have been noted in a number of organisms from similar environments to that of the Nepalese/Bangladeshi *S. spindale*, particularly from the Tibetan plateau, such as the plateau pika and Tibetan horse and antelope^45,46,52^. Evidence of similar adaptation has also been seen in *Schistosoma turkestanicum* inhabiting colder temperate climates^53^. This provides further evidence of the occurrence of a vicariance event separating the *S. spindale* linages. Thus, the data shown here supports a speciation event captured in process with the Nepal/Bangladesh cluster initially isolated by the up lift of the Nepalese plateau, and a Sri Lanka/Thailand/Malaysia cluster which most likely represents the *S. spindale* that radiated across South East Asia. However, further sampling, detailed assessment of host use and deeper genetic analyses is required in order to resolve the Nepal/Bangladesh and a Sri Lanka/Thailand/Malaysia clusters as distinct species.

Diversity and speciation events in *Schistosoma* lack detailed understanding, and this is especially true for the *S. indicum* group and its members. Thus, the prevalence of each species, information which is vital in planning effective treatment regimes, and the development and monitoring of control programmes, remains uncertain. Mitochondrial molecular markers have been shown to be vital in the identification of different schistosome species and for providing initial insights into the evolution and epidemiology of parasite populations. Although it is preferable to use complete mitochondrial genomes to address evolutionary issues within and between members of the *S. indicum* group, a paucity in published comparative molecular data exists. The *cox1*, *16* *s* and *12* *s* can provide a reliable species identification and initial insights in population biology of the *S. indicum* group that could inform control procedures and aid in assessing the impact of these parasites on food security and socioeconomic status of communities that depend on farmed animals, as well as the public health implications the *S. indicum* group poses to the people within these communities^54^.

## Materials and Methods

### Parasite material and DNA extraction

Adult *S. spindale* and *S. indicum* samples were collected from cattle and goat carcasses from local meat markets in Bangladesh as part of an ongoing study by the University of Agricultural and Veterinary Sciences, Bangladesh. Worms were identified morphologically based on the number of testes in males and ovaries in females and ten paired worms of each species were sent to Kingston University for genome analyses. Owing to low DNA concentrations from extractions of individual worms, pools of five males were used for DNA extraction for each species. Sample DNA was extracted using the Qiagen DNeasy blood and tissue extraction kit (Qiagen Inc.).

### Mitochondrial genome sequencing, assembly and annotation

The DNA samples were submitted to the Natural History Museum (London) sequencing facility to be sequenced using Illumina MiSeq next generation sequencing (NGS) (Illumina, Inc). Each DNA extract was indexed, and libraries prepared for NGS sequencing using the TrueSeq Nano DNA Sample Preparation Kit (Illumina, Inc). Both the *S. indicum* and *S. spindale* libraries were then run independently on a MiSeq Illumina sequencer (pipeline 1.9) yielding approximately 300 bp long paired-end reads. The resultant DNA sequence reads were then analysed using CLC genomics workbench 7.7.5 (https://www.qiagenbioinformatics.com/). As with other helminths such as *Paragonimus westermani*^55^, *Fasciolopsis buski*^56^, *Dirofilaria* species^57^ and *Trichuris* species^58^ reads were trimmed and adaptors removed using CLC, based on quality reports of length distribution, GC content and nucleotide contribution. The refined sequence reads were then used to assemble the mitochondrial genomes of *S. spindale* and *S. indicum* by mapping sequences to the published Sri Lankan *S. spindale* mitochondrial genome using parameters specified (Supplementary Fig. S2). There were no allelic forms identified in the mapped reads for either *S. indicum* or *S. spindale* respectively, and once assembled each novel genome sequence was locally realigned and the consensus was extracted for further analysis. Sequences were annotated based on homology with the Sri Lankan reference sequence, allowing the identification of individual genes and coding regions in the newly assembled genomes. MITOS was used to confirm genome annotation and produce secondary tRNA structures by uploading mitochondrial genomes to the server (http://mitos.bioinf.uni-leipzig.de/index.py). GenomeVX was used to create a circularized graphical representation of the mitochondrial genome (http://wolfe.ucd.ie/GenomeVx/) with positions of all genes specified based on the annotations from the genome assembly.

### Measuring divergence and estimating phylogenetic relationships of the *S. indicum* group

To assess the level of divergence between *S. indicum* and *S. spindale* the mitochondrial genomes of *S. spindale* and *S. indicum* from Bangladesh Sri Lankan *S. spindale* were aligned against each other using MUSCLE (https://www.ebi.ac.uk/Tools/msa/muscle/). Sequences for individual genes within the OXPHOS complexes were extracted from the alignments and pairwise average nucleotide divergence (Dxy) was calculated for both inter and intra species comparisons using DNASP v5.10^59^. As only a few complete mitochondrial genomes are available for the *S. indicum* group, it was not possible to accurately measure the occurrence of positive selection across the mitochondrial genome based on the ratio of synonymous and non-synonymous mutations to provide initial insight into the divergence between parasite species and isolates. Instead indirect measures were performed which would allow the comparison of differences between resultant protein peptide sequences. The proportion of amino acid replacements per gene was estimated for comparisons between *S. spindale* and *S. indicum*, as well as between both isolates of *S. spindale*. In both cases total number of amino acid differences between each protein was calculated in MEGA version X^60^, and then divided by the total number of amino acids within the protein produced by each gene. To assess the functional effect of each amino acid change Provean^26^, the protein variation effect analyser was used to assess differences both within and between species. By evaluating each protein sequence variation in an evolutionary context Provean^26^ predicts the likelihood of an amino acid substitution having a functional effect on the protein. The default confidence threshold of −2.5 was used as recommended by Choi and colleagues^15^ to determine if an amino acid replacement was likely to have a functional effect on the resultant protein. In order to identify the region of the protein that these amino acid replacements occurred within domains were predicted for each protein using TMHMM^26^ webserver (http://www.cbs.dtu.dk/services/TMHMM/) which employs a hidden Markov model to predicted transmembrane regions within a peptide sequence. Mitochondrial proteins are considered to have three separate domains: (i) those exposed to the inside of the mitochondria and interact directly with the mitochondrial matrix; (ii) those within the mitochondrial membrane providing structural support for the protein; (iii) those that are on the surface of the mitochondria and between the inner and outer membrane. Owing to their functional roles in regulating the electron transport chain it is considered that the OXPHOS complexes are under strong purifying selection, thus any amino acid replacements within the domains of such proteins could indicate alterations in function that has occurred as a result of positive selection. Subsequently, the proportion of putative function altering amino acid replacements were was also calculated between *S. spindale* and *S. indicum* as well as between the Bangladeshi and Sri Lankan *S. spindale* isolates.

Owing to the lack of complete mitochondrial genomes of other isolates of *S. spindale* and *S. indicum*, to resolve the phylogeny of the *S. indicum* group and disentangle inter and intra species relationship the *cox1*, *12* *s* and *16* *s* sequences were retrieved from the mitochondrial genomes and compared to published data representing species and isolates from across Asia (Supplementary Tables S2–4. Again, alignments were performed using MUSCLE and ML phylogenies were constructed for each gene using MEGA version X^60^. For each alignment nucleotide substitution models were also calculated using MEGA with the HKY + G + I indicated for the *cox1* alignment, the GTR + G for *16* *s* and the HKY + G for the *12* *s*. All maximum likelihood phylogenetic analyses used 1000 bootstrap replicates to provide nodal support and owing to its historical taxonomic association with the *S. indicum* group and utility in other studies^10,11^ *Schistosoma incognitum* was used as an outgroup to provide a root for each of the *S. indicum* group phylogenies. For comparison, phylogenetic relationships were also reconstructed using Bayesian inferences (BI) implemented through BEAST v.2.4.6 for each of the gene markers. The same substitution models were used as described above and for each of the genes an MCMC chain length of 10^7^ and a pre-burn-in of 10^6^ was used to construct the trees.

In order to estimate times of divergence within and between members of the *S. indicum* group Bayesian molecular clock analyses were also performed using BEAST with known *cox1* schistosome and trematode evolutionary rates of 0.035 ± 0.0071 substitutions per million^61,62^. In order to provide the most detail account of the divergence of the *S. indicum* group a full phylogenetic analyses of all *Schistosoma* species was performed with the the HKY + G + I substitution model again with an MCMC chain length of 10^7^ and a pre-burn-in of 10^6^. A strict clock approach was taken using conditions of the Yule model for speciation; an approach used in previous analysis of schistosome divergence^62^ and was calibrated based on published divergence time estimates between *S. mansoni* and *S. japonicum*, *S. mansoni* and *S. incognitum*, *S. mansoni* and *S. haematobium*^19^. Tracer 1.3 was used to check the effective sample size (ESS) was above the 200-threshold value as an indication of exploration of parameter space^63^. In order to summarise the posterior distribution of trees output by BEAST, the maximum clade credibility (MCC) tree was obtained using TreeAnnotator (part of the BEAST suite of programs) with a burn-in percentage of 10% and no posterior probability limit. The MCC tree was manipulated in FigTree v1.4.3 (http://tree.bio.ed.ac.uk/software/figtree/) where node ages and posterior probabilities could be assigned, and the visual appearance of the tree could be edited.

### Evolutionary analyses of *Schistosoma spindale*

Owing to the high levels of divergence identified between isolates of *S. spindale* in previous studies and across the mitochondrial genome in this current study, detailed analyses between *S. spindale* isolates were performed based on reanalyses of the few published DNA sequences from across Asia, whilst incorporating the sequence data generated in the present study. Initially, in order to identify the occurrence of shared mitochondrial haploytpes of *cox1*, *12* *s* and *16* *s* between localities, as well as to determine which haplotypes clustered together more readily, the most parsimonious haplotype networks were generated using TCS as implemented through PopART^64^. Although only a few sequences for each gene could be used the analyses provided initial insights into divergence of *S. spindale*, providing perspective of any underlying population structure.

Three methods of species delimitation were utilised to identify the number of species that were represented within the sampled sequences. Firstly, uncorrected *p*-distance values were obtained for both within and between species as a measure of percentage of divergence for both intra and inter species assessments. This was done for each marker sequence alignment using MEGA. This allowed the identification of pairwise comparisons between isolates which were 3% or higher, which is often used as the minimum level of divergence to differentiate between species using mitochondrial markers, especially *cox1* as the recognised barcoding gene^65^. Secondly, each of the alignments were submitted to the ABGD server^20^ to independently identify the number of molecular taxonomic units based on the total number of sequence clusters which grouped together within the data set. All parameters used within the ABGD analyses were assigned default settings and a 3% cut-off value, which is typically thought to be the species threshold used to identify the number of species within each group^66,67^. Finally, the Birky^21^ 4X ratio was applied to the *S. spindale* alignments. This final method of species delimitation compares the ratio of mean pairwise difference between two clades (K) and the mean pairwise difference within each clade (θ), with K/θ ≥ 4 indicating that the clades represent separate species. As two clades are being compared then there are two values produced for θ, as per the recommendations of Birky^21^, the larger value is used for the final K/θ calculation as this gives more conservative results, which are less likely to return false positives.

To identify the potential cause for the level of divergence, the impact of potential positive selection was assessed within and between *S. spindale* isolates using TreeSAAP^22^ to detect evidence of physiochemical property changes to the protein sequences. Sequences for *S. spindale cox1* were aligned and a Newick format ML tree was created in MEGA, from this TreeSAAP tested the sequences for alterations in physiochemical properties. The significance of such amino acid replacements was evaluated by a z-test which is also used to identify the type of selection acting upon the bases within a specific region specified within the sliding window. Only results from category 6–8 were used with high levels of significance (p < 0.001) as these categories show property changes that are having the most effect on protein function and could be related to adaptation. The same analysis was also performed on *cox1* samples of *S. indicum* and *S. cf. indicum*.

## Supplementary information

Supplementary Tables and Figures.
