# Author Correction: Divergence across mitochondrial genomes of sympatric members of the *Schistosoma indicum* group and clues into the evolution of *Schistosoma spindale*

**DOI:** 10.1038/s41598-021-81050-9

**Published:** 2021-01-07

**Authors:** Ben P. Jones, Billie F. Norman, Hannah E. Borrett, Stephen W. Attwood, Mohammed M. H. Mondal, Anthony J. Walker, Joanne P. Webster, R. P. V. Jayanthe Rajapakse, Scott P. Lawton

**Affiliations:** 1 Molecular Parasitology Laboratory, School of Life Sciences, Pharmacy and Chemistry, Kingston University London, Kingston Upon Thames, Surrey KT1 2EE UK; 2 Department of Life Sciences, Natural History Museum, Cromwell Road, London, SW7 5BD UK; 3 Department of Parasitology, Faculty of Veterinary Science, Bangladesh Agricultural University, Mymensingh, 2202 Bangladesh; 4 Centre for Emerging, Endemic and Exotic Diseases, Department of Pathobiology and Population Sciences, The Royal Veterinary College, University of London, Hatfeld, Hertfordshire AL9 7TA UK; 5 Faculty of Veterinary Medicine and Animal Science, Department of Veterinary Pathobiology, University of Peradeniya, Peradeniya, 20400 Sri Lanka

Correction to: *Scientific Reports* 10.1038/s41598-020-57736-x, published online 12 February 2020

The original version of this Article contained a typographical error in the spelling of the author R. P. V. Jayanthe Rajapakse, which was incorrectly given as P. R. V. Jayanthe Rajapakse.

As a result, the Author Contributions section was incorrect.

“B.P.J., A.J.W., J.P.W., P.R.V.J.R. and S.P.L. conceived the project. B.P.J., B.F.N., H.E.B., S.W.A. and S.P.L. performed initial assembly and bioinformatic analyses of *S. spindale* mitochondrial genomes. B.P.J., B.F.N. and S.P.L. performed initial assembly and analyses on the *S. indicum* mitochondrial genome. Detailed comparative genomic analyses and phylogenetics were performed by B.P.J. and S.P.L. S.W.A. and M.M.H.M. were responsible for collecting and providing parasite material. B.P.J., A.J.W., J.P.W., P.R.V.J.R. and S.P.L. were involved in writing the final text which was subsequently checked and approved by all authors. This work was undertaken as part of the MSc by Research of B.P.J.”

now reads:

B.P.J., A.J.W., J.P.W., R.P.V.J.R. and S.P.L. conceived the project. B.P.J., B.F.N., H.E.B., S.W.A. and S.P.L. performed initial assembly and bioinformatic analyses of *S. spindale* mitochondrial genomes. B.P.J., B.F.N. and S.P.L. performed initial assembly and analyses on the *S. indicum* mitochondrial genome. Detailed comparative genomic analyses and phylogenetics were performed by B.P.J. and S.P.L. S.W.A. and M.M.H.M. were responsible for collecting and providing parasite material. B.P.J., A.J.W., J.P.W., R.P.V.J.R. and S.P.L. were involved in writing the final text which was subsequently checked and approved by all authors. This work was undertaken as part of the MSc by Research of B.P.J.

These errors have now been corrected in the PDF and HTML versions of the Article, and in the accompanying Supplementary Information file.

