# Supplementary material for: Divergence across mitochondrial genomes of sympatric members of the Schistosoma indicum group and clues into the evolution of Schistosoma spindale
Source: Sci Rep. 2020 Feb 12;10:2480. doi: 10.1038/s41598-020-57736-x (PMC7015907; doi:10.1038/s41598-020-57736-x)
Supplement: Supplementary file 1 — Supplementary Tables and Figures. [file 41598_2020_57736_MOESM1_ESM.docx]

**Supplementary Material:**

**Divergence across mitochondrial genomes of sympatric members of the *Schistosoma indicum* group and clues into the evolution of *Schistosoma spindale***

Ben P. Jones^1^, Billie F. Norman^1^, Hannah E. Borrett^1^, Stephen W. Attwood^2^, Mohammed M. H. Mondal^3^, Anthony J. Walker^1^, Joanne P. Webster^4^, P. R. V. Jayanthe Rajapakse^5^ & Scott P. Lawton^1*^

1. Molecular Parasitology Laboratory, School of Life Sciences, Pharmacy & Chemistry, Kingston University London, Kingston Upon Thames, Surrey, KT1 2EE, UK.
2. Department of Life Sciences, Natural History Museum, Cromwell Road, London, SW7 5BD, UK.
3. Department of Parasitology, Faculty of Veterinary Science, Bangladesh Agricultural University, Mymensingh 2202, Bangladesh.
4. Centre for Emerging, Endemic and Exotic Diseases, Department of Pathobiology and Population Sciences, The Royal Veterinary College, University of London, Hatfield, Hertfordshire, AL9 7TA, United Kingdom.
5. Faculty of Veterinary Medicine and Animal Science, Department of Veterinary Pathobiology, University of Peradeniya, Peradeniya 20400, Sri Lanka.

***Corresponding author:** Molecular Parasitology Laboratory, School of Life Sciences, Pharmacy and Chemistry, Kingston University, Kingston upon Thames, Surrey KT1 2EE, UK. E-mail: s.p.lawton@kingston.ac.uk

**Table S1: List of amino acid replacements identified between *S. spindale* and *S. indicum* as well as between *S. spindale* from Bangladesh and Sri Lanka**. Replacements shown are predicted to have impact on the function of the resultant proteins of the mitochondria, position within specific domains are also highlighted.

| **Gene** | **Position** | ***S. spindale* Bangladesh** | ***S. spindale* Sri Lanka** | ***S. indicum*** | ***S. spindale* Bangladesh vs *S. indicum*** | ***S. spindale* Sri Lanka vs *S. indicum*** | ***S. spindale* Bangladesh vs *S. spindale* Sri Lanka** | **Domain** |
| --- | --- | --- | --- | --- | --- | --- | --- | --- |
| **ATP6** | 116 | P | P | N | -8.1 | -8.1 | 0 | Transmembrane helix |
| **ATP6** | 115 | R | R | H | -5 | -5 | 0 | Transmembrane helix |
| **ATP6** | 128 | V | M | G | -4.478 | 2.77 | -1.597 | Transmembrane helix |
| **ATP6** | 155 | M | M | V | -2.867 | -2.867 | 0 | Transmembrane helix |
| **COX1** | 212 | S | S | C | -2.692 | -2.692 | 0 | Intermembrane (Outside) |
| **COX2** | 180 | G | V | V | -8.56 | 0 | -8.56 | Mitochondrial matrix (Inside) |
| **COX2** | 177 | E | Q | E | 0 | -2.674 | -2.674 | Mitochondrial matrix (Inside) |
| **COX2** | 50 | S | S | L | -2.627 | -2.627 | 0 | Transmembrane helix |
| **Cytb** | 251 | P | S | F | -6.506 | 1.868 | -4.585 | Intermembrane (Outside) |
| **Cytb** | 196 | S | S | L | -3.299 | -3.299 | 0 | Mitochondrial matrix (Inside) |
| **Cytb** | 6 | N | I | I | -3.025 | 0 | -3.025 | Mitochondrial matrix (Inside) |
| **Cytb** | 124 | H | R | H | 0 | -3.643 | -3.399 | Mitochondrial matrix (Inside) |
| **Cytb** | 294 | P | P | V | -4.616 | -4.616 | 0 | Transmembrane helix |
| **Cytb** | 302 | Y | S | S | -3.537 | 0 | -3.537 | Transmembrane helix |
| **Cytb** | 292 | W | W | L | -3.402 | -3.402 | 0 | Transmembrane helix |
| **Cytb** | 177 | A | A | V | -2.837 | -2.837 | 0 | Transmembrane helix |
| **Cytb** | 222 | F | F | L | -2.808 | -2.808 | 0 | Transmembrane helix |
| **Cytb** | 224 | L | W | W | -2.727 | 0 | -2.727 | Transmembrane helix |
| **NAD1** | 204 | L | L | G | -6.554 | -6.554 | 0 | Mitochondrial matrix (Inside) |
| **NAD1** | 205 | V | V | G | -5.985 | -5.985 | 0 | Mitochondrial matrix (Inside) |
| **NAD1** | 180 | I | I | N | -3.985 | -3.985 | 0 | Transmembrane helix |
| **NAD1** | 119 | L | L | A | -3.153 | -3.153 | 0 | Transmembrane helix |
| **NAD2** | 196 | W | W | S | -9.745 | -9.745 | 0 | Transmembrane helix |
| **NAD2** | 242 | Y | Y | L | -2.869 | -2.869 | 0 | Transmembrane helix |
| **NAD3** | 92 | Y | L | L | -3.482 | 0 | -3.482 | Transmembrane helix |
| **NAD3** | 71 | F | F | L | -3.267 | -3.267 | 0 | Transmembrane helix |
| **NAD4** | 350 | V | V | S | -2.926 | -2.926 | 0 | Intermembrane (Outside) |
| **NAD4L** | 9 | G | G | S | -4.689 | -4.689 | 0 | Transmembrane helix |
| **NAD4L** | 65 | M | I | I | -3.429 | 0 | -3.429 | Transmembrane helix |
| **NAD5** | 96 | G | G | C | -4.106 | -4.106 | 0 | Intermembrane (Outside) |
| **NAD5** | 183 | L | L | F | -3.443 | -3.443 | 0 | Mitochondrial matrix (Inside) |
| **NAD5** | 169 | V | V | Y | -3.544 | -3.473 | 0 | Transmembrane helix |
| **NAD5** | 242 | G | G | S | -2.842 | -2.908 | 0 | Transmembrane helix |
| **NAD5** | 58 | S | G | C | -1.635 | -2.57 | 0.886 | Transmembrane helix |
| **NAD5** | 445 | F | V | G | -1.343 | -2.716 | 1.328 | Transmembrane helix |
| **NAD5** | 121 | F | S | V | 0.654 | -2.825 | 3.453 | Transmembrane helix |
| **NAD6** | 153 | Y | Y | N | -5.775 | -5.811 | 0 | Mitochondrial matrix (Inside) |
| **NAD6** | 154 | F | F | C | -3.377 | -3.213 | 0 | Mitochondrial matrix (Inside) |

**Table S2: *Schistosoma indicum* group *cox1* accession numbers**. Table contains accession numbers or origins of sequences used for the construction of *cox1* phylogenies in Figure 4.

| **Species** | **Accession** | **Location** |
| --- | --- | --- |
| *S. spindale* | KR607228.1 | Nepal |
| *S. spindale* | KR607227.1 | Nepal |
| *S. spindale* | KR607226.1 | Nepal |
| *S. spindale* | KR607225.1 | Nepal |
| *S. spindale* | KR607224.1 | Nepal |
| *S. spindale* | KR607223.1 | Nepal |
| *S. spindale* | KR607222.1 | Nepal |
| *S. spindale* | DQ157223.1 | Sri Lanka |
| *S. spindale* | AY157203.1 | Sri Lanka |
| *S. spindale* | Agatsuma *et al*., 2002 | Malaysia |
| *S. spindale* | This study | Bangladesh |
| *S. cf. indicum* | KR607221.1 | Nepal |
| *S. cf. indicum* | KR607220.1 | Nepal |
| *S. cf. indicum* | KR607219.1 | Nepal |
| *S. cf. indicum* | KR607218.1 | Nepal |
| *S. cf. indicum* | KR607217.1 | Nepal |
| *S. cf. indicum* | KR607216.1 | Nepal |
| *S. cf. indicum* | KR607215.1 | Nepal |
| *S. cf. indicum* | KR607214.1 | Nepal |
| *S. cf. indicum* | KR607213.1 | Nepal |
| *S. indicum* | AY157204.1 | Bangladesh |
| *S. indicum* | This study | Bangladesh |
| *S. indicum* | Agatsuma *et al*., 2002 | Bangladesh |
| *S. nasale* | KR607232.1 | Nepal |
| *S. nasale* | KR607231.1 | Nepal |
| *S. nasale* | KR607230.1 | Nepal |
| *S. nasale* | KR607229.1 | Nepal |
| *S. nasale* | AY157205.1 | Sri Lanka |
| *S. nasale* | Agatsuma *et al*., 2002 | Sri Lanka |
| *S. incognitum* | JQ408708.1 | India |
| *S. incognitum* | AY157201.1 | Thailand |
| *S. incognitum* | Agatsuma *et al*., 2002 | Thailand |

**Table S3: *Schistosoma indicum* group *16s* accession numbers.** Table contains accession numbers or origins of sequences used for the construction of *16s* phylogenies in Figure 4.

| **Species** | **Accession** | **Location** |
| --- | --- | --- |
| *S. spindale* | KR423842.1 | Nepal |
| *S. spindale* | KR423841.1 | Nepal |
| *S. spindale* | KR423840.1 | Nepal |
| *S. spindale* | KR423839.1 | Nepal |
| *S. spindale* | EF534290.1 | Thailand |
| *S. spindale* | EF534289.1 | Sri Lanka |
| *S. spindale* | DQ157223.1 | Sri Lanka |
| *S. spindale* | EF534288.1 | Bangladesh |
| *S. spindale* | This study | Bangladesh |
| *S. cf. indicum* | KR423838.1 | Nepal |
| *S. cf. indicum* | KR423837.1 | Nepal |
| *S. cf. indicum* | KR423836.1 | Nepal |
| *S. cf. indicum* | KR423835.1 | Nepal |
| *S. cf. indicum* | KR423834.1 | Nepal |
| *S. cf. indicum* | KR423833.1 | Nepal |
| *S. cf. indicum* | KR423832.1 | Nepal |
| *S. indicum* | EF534284.1 | Bangladesh |
| *S. indicum* | This study | Bangladesh |
| *S. nasale* | KR423844.1 | Nepal |
| *S. nasale* | KR423843.1 | Nepal |
| *S. incognitum* | EF534286.1 | Indonesia |
| *S. incognitum* | EF534287.1 | Thailand |
| *S. incognitum* | EF534285.1 | Bangladesh |

**Table S4: *Schistosoma indicum* group *12s* accession numbers.** Table contains accession numbers or origins of sequences used for the construction of *12s* phylogenies in Figure 4.

| **Species** | **Accession** | **Location** |
| --- | --- | --- |
| *S. spindale* | KR607258.1 | Nepal |
| *S. spindale* | KR607257.1 | Nepal |
| *S. spindale* | KR607256.1 | Nepal |
| *S. spindale* | KR607255.1 | Nepal |
| *S. spindale* | KR607254.1 | Nepal |
| *S. spindale* | KR607253.1 | Nepal |
| *S. spindale* | KR607252.1 | Nepal |
| *S. spindale* | KR607251.1 | Nepal |
| *S. spindale* | KR607250.1 | Nepal |
| *S. spindale* | AF465920.1 | Thailand |
| *S. spindale* | AF465919.1 | Thailand |
| *S. spindale* | EF534283.1 | Thailand |
| *S. spindale* | EF534282.1 | Sri Lanka |
| *S. spindale* | DQ157223.1 | Sri Lanka |
| *S. spindale* | EF534281.1 | Bangladesh |
| *S. spindale* | This study | Bangladesh |
| *S. cf. indicum* | KR607249.1 | Nepal |
| *S. cf. indicum* | KR607248.1 | Nepal |
| *S. cf. indicum* | KR607247.1 | Nepal |
| *S. cf. indicum* | KR607246.1 | Nepal |
| *S. cf. indicum* | KR607245.1 | Nepal |
| *S. cf. indicum* | KR607244.1 | Nepal |
| *S. cf. indicum* | KR607243.1 | Nepal |
| *S. cf. indicum* | KR607242.1 | Nepal |
| *S. cf. indicum* | KR607241.1 | Nepal |
| *S. cf. indicum* | KR607240.1 | Nepal |
| *S. cf. indicum* | KR607239.1 | Nepal |
| *S. cf. indicum* | KR607238.1 | Nepal |
| *S. cf. indicum* | KR607237.1 | Nepal |
| *S. cf. indicum* | KR607236.1 | Nepal |
| *S. cf. indicum* | KR607235.1 | Nepal |
| *S. cf. indicum* | KR607234.1 | Nepal |
| *S. cf. indicum* | KR607233.1 | Nepal |
| *S. indicum* | EF534276.1 | Bangladesh |
| *S. indicum* | This study | Bangladesh |
| *S. nasale* | KR607262.1 | Nepal |
| *S. nasale* | KR607261.1 | Nepal |
| *S. nasale* | KR607260.1 | Nepal |
| *S. nasale* | KR607259.1 | Nepal |
| *S. nasale* | EF534280.1 | Bangladesh |
| *S. incognitum* | EF534278.1 | Indonesia |
| *S. incognitum* | EF534279.1 | Thailand |
| *S. incognitum* | AF465915.1 | Thailand |
| *S. incognitum* | EF534277.1 | Bangladesh |

**Table S5: Accession numbers for species used in the molecular clock analyses.** Table contains accession numbers or origins of sequences used for the construction of molecular clock seen in Figure 5.

| **Species** | **Accesssion** |
| --- | --- |
| *T. regenti* | AY157190.1 |
| *B. nairi* | JQ975007.1 |
| *S. douthitti* | AY157193.1 |
| *S. sinensium* | AY157197.1 |
| *S. japonicum* | KU196417.1 |
| *S. malayensis* | AY157198.1 |
| *S. mekongi* | AY157199.1 |
| *Schistosoma sp.* Uganda | AY197348.1 |
| *S. hippopotami* | AY197346.1 |
| *S. edwardiense* | AY197347.1 |
| *S. incognitum* | AY157201.1 |
| *S. turkestanicum* | AY157200.1 |
| *Schistosoma sp.* RD-2016 | KT022105.1 |
| *S. mansoni* | AF101196.1 |
| *S. rodhaini* | AY157202.1 |
| *S. nasale* | AY157205.1 |
| *S. indicum* | This study |
| *S. cf. indicum* | KR607221.1 |
| *S. spindale* | This study |
| *S. spindale* | AY157203.1 |
| *S. margrebowiei* | AY157206.1 |
| *S. mattheei* | AY157211.1 |
| *S. intercalatum* | AJ519515.1 |
| *S. kisumuensis* | FJ897159.1 |
| *S. leiperi* | AY157207.1 |
| *S. haematobium* | AY157209.1 |
| *S. guineensis* | AJ519517.1 |
| *S. bovis* | AY157212.1 |
| *S. curassoni* | AY157210.1 |

**Supplementary Figures**


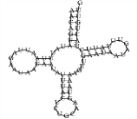

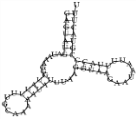

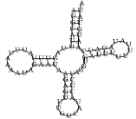

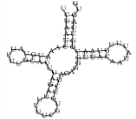

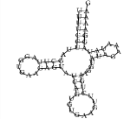

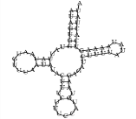

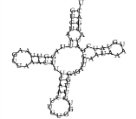

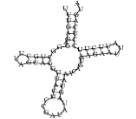

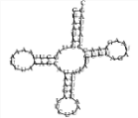

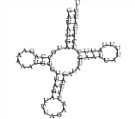

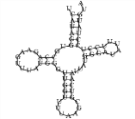

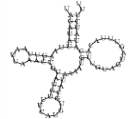

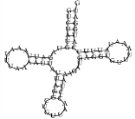

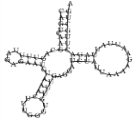

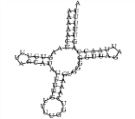

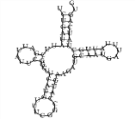

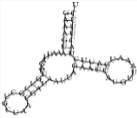

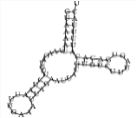

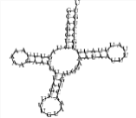

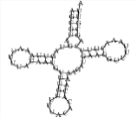

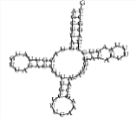

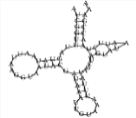


**A**

**C**

**D**

**E**

**R**

**I**

**H**

**G**

**F**

**N**

**P**

**Q**

**V**

**W**

**Y**

**T**

**L1**

**L2**

**M**

**S1**

**S2**

**K**

**A) *Schistosoma spindale* Sri Lanka**


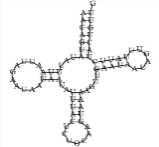

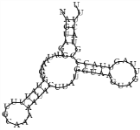

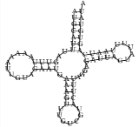

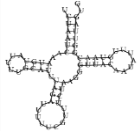

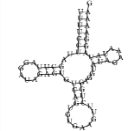

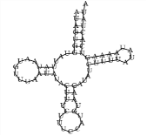

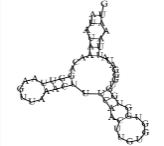

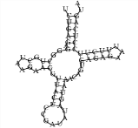

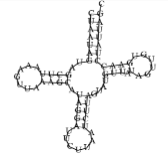

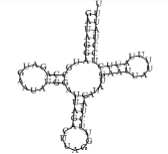

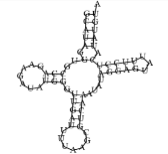

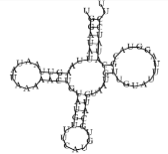

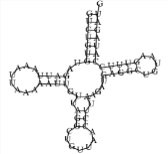

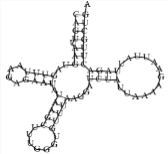

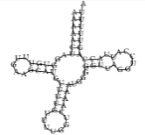

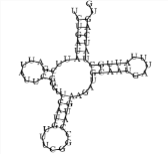

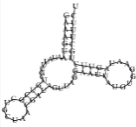

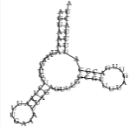

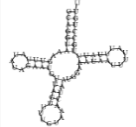

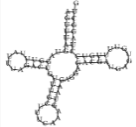

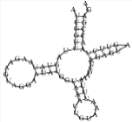

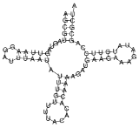


**A**

**C**

**D**

**E**

**R**

**I**

**H**

**G**

**F**

**N**

**P**

**Q**

**V**

**W**

**Y**

**T**

**L1**

**L2**

**M**

**S1**

**S2**

**K**


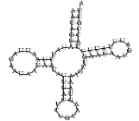

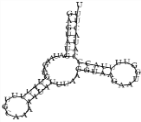

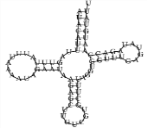

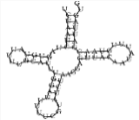

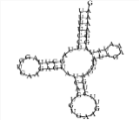

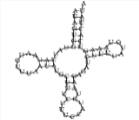

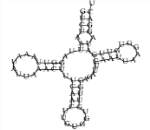

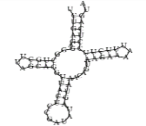

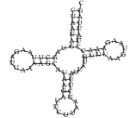

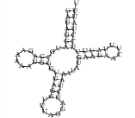

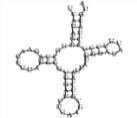

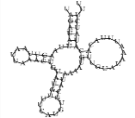

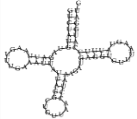

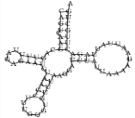

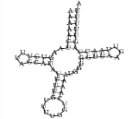

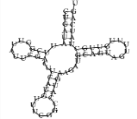

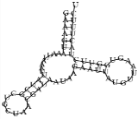

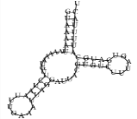

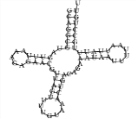

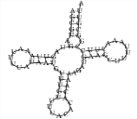

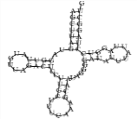

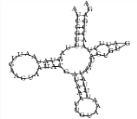


**A**

**C**

**D**

**E**

**R**

**I**

**H**

**G**

**F**

**N**

**P**

**Q**

**V**

**W**

**Y**

**T**

**L1**

**L2**

**M**

**S1**

**S2**

**K**

**B) *Schistosoma spindale* Bangladesh**

**C) *Schistosoma indicum* Bangladesh**

**Figure S1 Comparison of tRNA structures between *S. spindale* and *S. indicum*.** tRNA secondary structures created in MITOS showing the similarity in structure shared between the two closely related species. In *S. indicum* the tRNA structures that appear to be altered are highlighted.

Raw reads imported into CLC and paired. Failed reads removed, paired distances set at 100-1200. Quality report produced.

Reads trimmed with settings: Quality-0.01, Ambiguities-0 Length-<50. Quality report produced.

Adapters trimmed from reads, Adapters used TruSeq universal & TruSeq adapter index-1. Reverse compliment of adapters trimmed using alignment score (Mismatch-2, Gap cost-1), Match fresh-hold (Internal matches- minimum 10, End matches- minimum 4). Any reads <50 discarded. Quality report produced.

Reads trimmed to satisfy quality reports- *S. spindale* trimmed of 15base pairs (bp) 5’ and 50bp 3’. 1^st^ *S. indicum* set trimmed of 10bp on 5’ and 5bp on3’. 2^nd^ *S. indicum* set trimmed of 30bp from 5’ and 3’

*S. indicum* sets merged.

Reads assembled using reference genomes. *S. spindale* (DQ157223.1) for mitochondrial genome and *S. mansoni* (HE601624 - 31) for nuclear genome. Parameters: No masking, Mismatch cost-2, Insertion cost-3, Deletion cost- 3, Length fraction- 0.5, Similarity fraction- 0.5. Paired distances automatically detected and Non-specific matches were randomly mapped.

Local realignment carried out on whole genome/chromosomes. Annotations copied from reference genomes to consensus sequence.

Consensus sequences exported in FASTA format

**Figure S2 Genome assembly method in CLC genomics workbench.** Flow chart detailing the genome assembly process in CLC genomics workbench v.7.5.5. including all parameters, data sets and reference sequences used. Before and after results for trimming can be seen in Supplementary figures 1–3.
